# The Influence of Weld Interface Characteristics on the Bond Strength of Collision Welded Aluminium–Steel Joints

**DOI:** 10.3390/ma17153863

**Published:** 2024-08-04

**Authors:** Stefan Oliver Kraus, Johannes Bruder, Peter Groche

**Affiliations:** Institute for Production Engineering and Forming Machines, TU Darmstadt, D-64287 Darmstadt, Germany; stefan.kraus@ptu.tu-darmstadt.de (S.O.K.); johannes.bruder@ptu.tu-darmstadt.de (J.B.)

**Keywords:** collision welding, impact welding, electromagnetic pulse welding, welding window, metallographic weld zone, multi-material construction, lightweight concepts, weight reduction, emission reduction

## Abstract

Collision welding is a promising approach for joining conventional materials in identical or dissimilar combinations without heat-related strength loss, thereby opening up new lightweight potential. Widespread application of this technology is still limited by an insufficient state of knowledge with respect to the underlying joining mechanisms. This paper applies collision welding to a material combination of DC04 steel and EN AW 6016 aluminium alloy. Firstly, the welding process window for the combination is determined by varying the collision speed and the collision angle, the two main influencing variables in collision welding, using a special model test rig. The process window area with the highest shear tensile strength of the welded joint is then determined using shear tensile tests and SEM images of the weld zone. The SEM investigations reveal four distinct metallographic structures in the weld zones, the area fractions of which are determined and correlated with collision angle and shear tensile strength.

## 1. Introduction

Mobility continues to be seen as an essential part of modern society, enabling people to access fundamental areas of society such as education, healthcare and the labour market. The concept of mobility covers a broad spectrum from private transport to public transport, the transport and logistics sector and basic infrastructure [1]. In 2022, 21% of global CO_2_ emissions were attributable to the transport sector, 48% of which was caused by private transport [2,3]. In addition to CO_2_ emissions from combustion vehicles, both electric and conventional combustion vehicles emit harmful particulate matter in the form of brake dust and tyre abrasion as so-called “non-exhaust particle emissions”. In total, up to ten percent of the microplastics released into the world’s oceans can be attributed to tyre abrasion from vehicles. The amount of non-exhaust particulate matter emitted per vehicle exceeds the amount of exhaust particulate matter emitted by modern combustion vehicles, which is why the future Euro 7 emission standard in Europe will also regulate “non-exhaust particle emissions” for the first time [4,5,6,7,8,9,10,11,12].

One way to reduce these emissions is to reduce vehicle mass [13,14,15]. For example, for small cars with internal combustion engines, a reduction in vehicle mass of 100 kg results in a reduction in CO_2_ emissions of 10 gCO_2_/km [13]. Vehicle weight also has a significant impact on non-exhaust particulate matter emissions. For example, the additional weight of 318 kg in an electric car compared to a comparable petrol car leads to an increase of up to 22% in non-exhaust particulate matter emissions. It is clear, how important weight-optimised design is for the environment [16]. These lightweighting goals can be achieved with the materials commonly used in automotive engineering, aluminium and steel, through the use of multi-material construction, particularly in conjunction with high-strength material variants [17]. In multi-material components, the different materials and alloys are combined to meet structural performance and weight requirements. Due to the different properties of the materials to be joined, fusion welding processes such as tungsten inert gas welding (TIG) or laser beam welding reach their limits in multi-material construction. The main reason for this is the different melting temperatures of the different materials [18,19]. If a material bond is possible at all, the melting of the materials leads to the formation of intermetallic phases in the weld area. The associated loss of ductility in the brittle metal joints restricts the potential of the used materials [20,21]. In order to exploit the existing lightweight potential of multi-material construction to the maximum, a joining process is therefore required that allows the material bonding of steels with aluminium alloys with the lowest possible loss of strength.

In this area, the solid state welding or collision welding process group opens up new opportunities. The joining mechanism is based on the application of sufficiently high pressure to join similar and dissimilar material combinations without active heat input. The formation of intermetallic phases in the joint zone is avoided [20,21,22]. The process window of the respective material combinations in which joining is possible is spanned by two variables, collision speed and collision angle. Depending on the collision angle, the process window is divided into three sub-areas with different joining mechanisms. These are the solid-phase joining region, the liquid-phase joining region and a hybrid region. Both joining mechanisms occur in the hybrid region [23]. Electromagnetic pulse welding (EMPW) exploits the advantages of collision welding in an industrial production environment [24,25]. In EMPW, one joining partner is accelerated to a high speed by a strong electromagnetic field and welded to the second joining partner through the subsequent high-speed collision [19,22]. Due to transient phenomena, the collision speed and the collision angle change dynamically during the course of the collision in the EMPW process. The transient process conditions influence the weld properties [26,27]. The process design in EMPW is currently exclusively iterative, which is costly and time-consuming on the one hand and leads to uncertainties in series production on the other hand [28]. For future optimised design of EMPW processes, it is therefore necessary to know the critical angle profile for the joint design during the collision in order to best influence the achievable strength properties of the joint in a robust way.

In this work, a mechanical collision welding test rig is used to investigate the formation of the joint zone and the joining mechanisms occurring in it at different collision angles for the material combination DC04 with EN AW 6016-T4. The aim is to identify the ideal angle range for a joint to determine the strength values of the welded joint as a function of collision angle and speed. In contrast to the previous work, in which the strength of the welded joint was determined and the weld zone was observed at individual points using microscope images, here, for the first time, the area ratios of the joint structures are determined over the entire length of the weld zone using SEM images to correlate the determined ratios with the tensile shear strength. This should form the basis for the future development of an analytical approach that can be used to calculate the strength of EMPW joints on the basis of simulated angle curves. The equipment used and the tests and investigations carried out are presented in the following section.

## 2. Materials and Methods

The tests in this work are carried out on a model test rig for collision welding; see the functional description below. The specimens used are taken from aluminium sheets (EN AW 6016-T4, thickness: 1.5 mm, tensile strength R_m_: 235 N/mm^2^) and steel sheets (DC04, thickness: 2.0 mm, tensile strength R_m_: 310 N/mm^2^) by shear cutting. For each material, three tensile tests were performed on a Zwick Roell 100 (Zwick Roell, Ulm, Germany) combined tensile and compression testing machine to determine tensile strength in accordance with DIN 50125.

**Figure 1:**
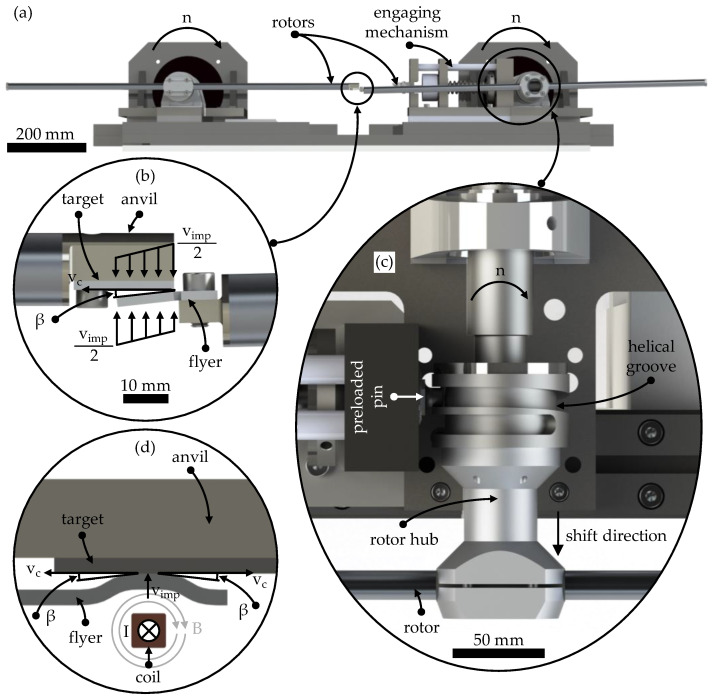
(**a**) The model test rig consists of two synchronised rotating rotors and an engaging mechanism. (**b**) The specimens are mounted at the end of each rotor. The target specimen is supported at the rear by an anvil. The flyer specimen is pre-bent to set the collision angle *β*. (**c**) In the engaging mechanism, a pin is preloaded by an electromagnet and a spring. When the electromagnet is deactivated, the pin engages in the helical groove and the entire rotor hub is shifted within one revolution so that both rotors are in one plane and the specimens collide. (**d**) Process setup for EMPW according to [33]. The initially flat flyer is accelerated by the electric field of the coil towards the stationary target, which is also supported on its back. The collision speed *v_imp_* and angle *β* change during the collision.

The model test rig for collision welding, developed at the Institute for Production Engineering and Forming Machines (PtU) at the Technical University of Darmstadt, allows the collision welding process to be investigated over the time profile of the collision with constant and precisely adjustable collision parameters thanks to its fully mechanical design. At the same time, it offers good observability of the process and the process phenomena. Figure 1a shows the construction of the model test rig. The specimens to be welded (collision area: length × width: 12.5 mm × 12 mm) are mounted at the ends of the two rotors rotating in the same direction. The EN AW 6016-T4 flyer specimen is pre-bent to set the collision angle *β*. The DC04 target is flat and is additionally supported by an anvil; see Figure 1b. To eliminate any influence on the test results from lubricant or corrosion inhibitor residues on the specimen surfaces, the specimens are cleaned with acetone immediately prior to testing. Both samples are accelerated in the model test rig to half of the desired impact velocity *v_imp_* by rotating the rotors at a specified speed. The collision speed *v_imp_* is varied in five steps from 262 m/s, 279 m/s, 305 m/s, 331 m/s and 349 m/s. For each collision speed, the collision angle *β* is varied to determine the welding process window. The collision angle is varied between 3.5° and 13.5°. During the collision of flyer and target, the collision front moves along the joining surface at the collision point velocity *v_c_*. The following trigonometric relationship expresses the dependence between the variables [23].

(1) $$\tan\beta =\frac{v_{imp}}{v_{c}}$$

As the rotors cannot reach the required speed within one revolution, both rotors initially accelerate axially offset from each other. Once the required speed has been reached, one rotor is shifted along its axis of rotation by means of a sliding rotor hub, as shown in Figure 1c. This is achieved by a preloaded pin engaging a helical groove in the rotor hub. Due to the predetermined kinematics of this cam gear, the axial offset of 15 mm between the two rotors is achieved within one revolution and the specimens collide exactly in the centre between the pivot points of the two rotor axes. During the collision or welding process, the flyer sample is separated from the flyer rotor by being torn off at a predetermined breaking point. The welded samples remain on the target rotor until the rotors come to a standstill.

Figure 1d shows the electromagnetic pulse welding (EMPW) process described in the previous section. In contrast to the model test rig, in EMPW, the flyer is accelerated towards the target by an electromagnetic field. When using a combination of aluminium and steel, aluminium is always used as the flyer material. This is due to its much higher electrical conductivity compared to steel [29]. The target, in this case, the steel sheet, is supported in the EMPW so that the kinetic energy of the flyer is fully utilised for welding during the collision process and the steel target is not set in motion by the collision. During this collision process, the flyer rolls sideways to the coil on the target, causing the dynamic change in collision angle *β* and collision velocity *v_imp_* described above. The mechanical design of the model test rig thus allows the transient EMPW collision-welding process to be studied at discrete points, each with a constant collision angle and velocity, and their influence on the properties of the welded joint. The aluminium alloy is used as the flyer and the steel target is supported on the back by the anvil in order to reproduce the EMPW process for the material combination mentioned as closely as possible with the model test rig and to be able to investigate the process behaviour.

In particular, the constant collision position of the two samples on the model test rig allows for good observation of the process. High-speed optical observation is performed using a *hsfc pro* image intensifier camera from *PCO* (Kelheim, Germany) and a *Milvus Macro 100 mm f2.0* macro lens from *Zeiss* (Oberkochen, Germany). The camera allows exposure times of <20 ns and the acquisition of up to eight images per collision. The brightness of the high-speed images is ensured by a *CAVILUX Smart* illumination laser from *Cavitar* (power: 400 W, wavelength: 640 nm, Tampere, Finland). The collision angle *β* is determined from the high-speed images using a *MATLAB* script (*MathWorks*, version: 2022b, Natick, Massachusetts, USA) based on edge detection. Detailed information on process monitoring and image processing of the high-speed images can be found in [30,31,32].

**Figure 2:**
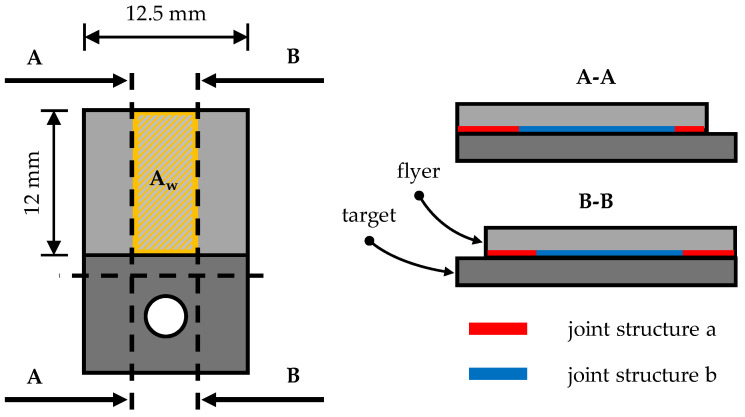
Graphic visualisation of the specimen cutting process and exemplary representation of different joint structures along the length of the weld zone.

In order to analyse the weld zone, the mounting face and lateral edge areas of the collision welded specimens are first cut off using a wet cut-off grinder in order to remove any effects of specimen manufacture in the edge areas of the weld and to shorten the overall length of the specimen for microscopic examination; see Figure 2. The samples are cut to a width of 8 mm ± 1 mm and a length of 14 ± 1 mm. The cut surfaces of the specimens are then ground on grinding and polishing machines with SiC paper (grit size P320, P400, P600, P800, P1200, P2500 and P4000) and polished with diamond suspension (3 µm and 1 µm). During grinding, the sample width is further reduced to 5 ± 1 mm. The weld zone is examined microscopically using a Phenom ProX scanning electron microscope (SEM), manufactured by Phenom-World (Waltham, MA, USA). If different joint structures occur along the length of the weld zone, the lengths of the corresponding areas are measured with the microscope in order to analyse correlations between the occurring joint structures and the shear tensile strength of the welded joint.

The tensile shear strength of the welded joint is determined using a fixture developed at PtU on a Zwick Roell 100 combined tensile and compression testing machine. In the fixture, the specimens (lateral edge areas and mounting face separated) are clamped between a clamping element and a hardened steel shear plate. The testing machine then moves at a test speed of 0.1 mm/s and applies shear to the welded joint via a second shear plate until the joint fails. All moving parts of the fixture are guided on bronze sliding plates with embedded graphite to reduce the effect of friction on the tensile shear test results. The repeatability of tensile shear tests depends on the overall Zwick Roell 100 combined tensile and compression testing machine with an integrated force sensor. For further information on the method used to test joint strength in the tensile shear test, see [23].

## 3. Results

### 3.1. Welding Process Window

**Figure 3:**
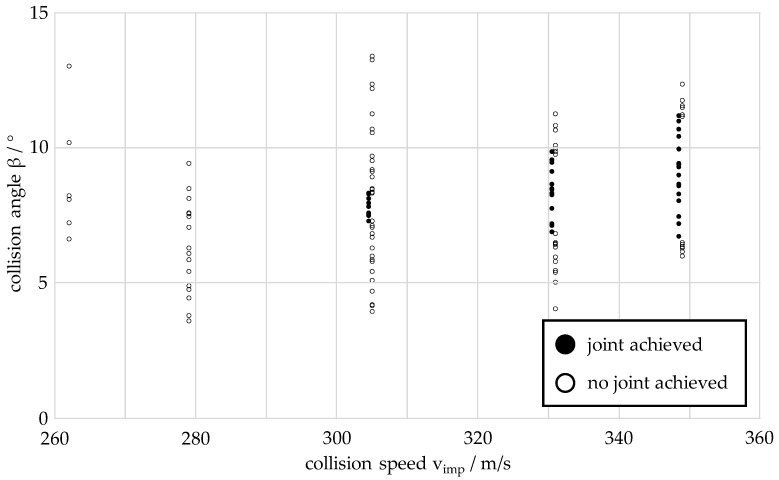
Welding process window of the material combination EN AW 6016-T4 (flyer, thickness: 1.5 mm) and DC04 (target, thickness: 2.0 mm).

This section presents the welding process window determined by collision welding tests for the material combination of aluminium EN AW 6016-T4 as the flyer and steel DC04 as the target. The materials have sheet thicknesses of 1.5 mm (EN AW 6016-T4) and 2.0 mm (DC04). As explained in the previous section, the collision welding tests are carried out at the five collision speeds *v_imp_* of 262 m/s, 279 m/s, 305 m/s, 331 m/s and 349 m/s. The process window determined is shown in Figure 3. In determining the process window, a collision weld test is defined as joined if the two specimens cannot be manually separated after the test. To improve the clarity of the process window diagram, the tests in which a joint is achieved are shifted 2 m/s to the left on the collision velocity axis.

At the two lowest collision speeds of 262 m/s and 279 m/s, there are still no bonds between the two materials. The first bonds between the two materials occur at a collision speed of 305 m/s. The lower limit angle is 7.3° and the upper limit angle is 8.3°. The angular range of the process window at this collision speed is 1.0°. At a collision speed of 331 m/s, a smaller lower limit angle of 6.8° is determined, while the upper limit angle increases to 9.8°. Even at the highest collision speed analysed, 349 m/s, there is again a slight reduction in the lower limit angle to 6.5° and a greater increase in the upper limit angle to 11.2°. The welding process window identified shows a clear separation of the areas where there is a bond or no bond between the two materials, both at the lower and upper limit angles.

### 3.2. Tensile Shear Strength of Welded Joints in the Center of the Welding Window at 305 m/s, 331 m/s and 349 m/s

Once the process window has been determined, it is first investigated whether the tensile shear strength of the welded joints of the material combination that can be produced by collision welding is dependent on the respective collision speed or whether the tensile shear strength values of the welded joints are independent of the collision speed. The tensile shear strength is compared at three different collision speeds (305 m/s, 331 m/s and 349 m/s). Only specimens that are in the centre of the process window at each speed are used for this comparison.

However, the collision angle can only be preset in 0.5° increments by pre-bending the flyer samples. In addition, due to the dynamics of the process in the model test rig, elastic deformations of the rotors occur, e.g., due to the engagement process, air resistance and aerodynamic turbulence in the rotor protection housing. These result in a deviation of the actual measured collision angle from the preset collision angle. For these reasons, a larger number of samples are welded at each of the three collision speeds and then three samples are selected for each collision speed that are within a range of ±0.3° around the mean collision angle of the process window. For the process window shown in Figure 3, the mean collision angle at a collision speed of 305 m/s is 7.8°. At 331 m/s the angle is 8.3° and at 349 m/s 8.8°.

**Figure 4:**
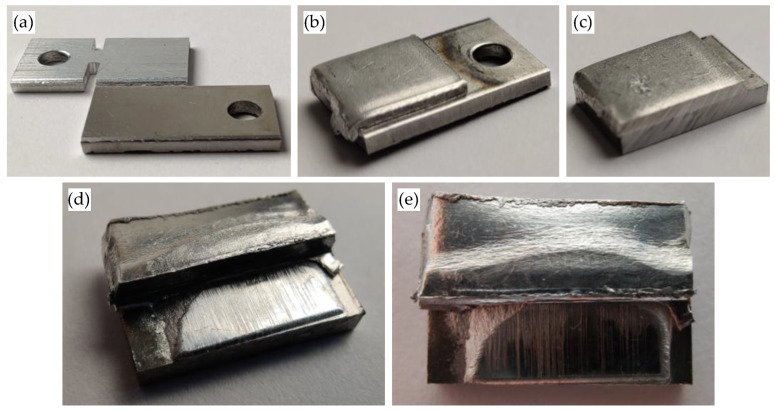
Images of the welded specimens (*v_imp_*: 349 m/s, *β*: 9,2°, welding direction: left to right) of steel DC04 (bottom) and aluminium EN AW 6016-T4 (top): (**a**) flyer and target in initial state; (**b**) welded specimens, with traces of ejected jet and particle cloud in area of mounting hole; (**c**) cut sample (grinding and polishing still to be carried out); (**d**,**e**) macro-image of welded specimens after tensile shear test, shear or sliding failure at the beginning and end of the weld zone and shear failure in the aluminium material in the middle of the weld zone.

The values obtained show an increase in tensile shear strength with increasing collision speed. At 305 m/s, the average tensile shear strength is 22 N/mm^2^, and at 331 m/s, it is 55 N/mm^2^. At both collision speeds, the welded joints shear or slide against each other in the weld zone. A strength of 130 N/mm^2^ is determined for the specimens welded at a collision speed of 349 m/s. Compared to the other specimens, these show a combined failure pattern when the maximum shear strength is reached. Along the length of the weld zone, there are areas where the specimens are separated by shear in the weld zone or slide against each other (similar to the specimens at 305 m/s and 331 m/s) and areas where shear occurs within the aluminium material, which is less strong than steel. The strength of the welded joint in these areas is therefore greater than the tensile shear strength of the aluminium material. Viewed over the length of the weld zone, shear failure in the aluminium material occurs predominantly in the middle, whereas shear or sliding in the weld zone occurs predominantly at the beginning and end; see Figure 4d,e.

### 3.3. Joint Structures in the Weld Zone

**Figure 5:**
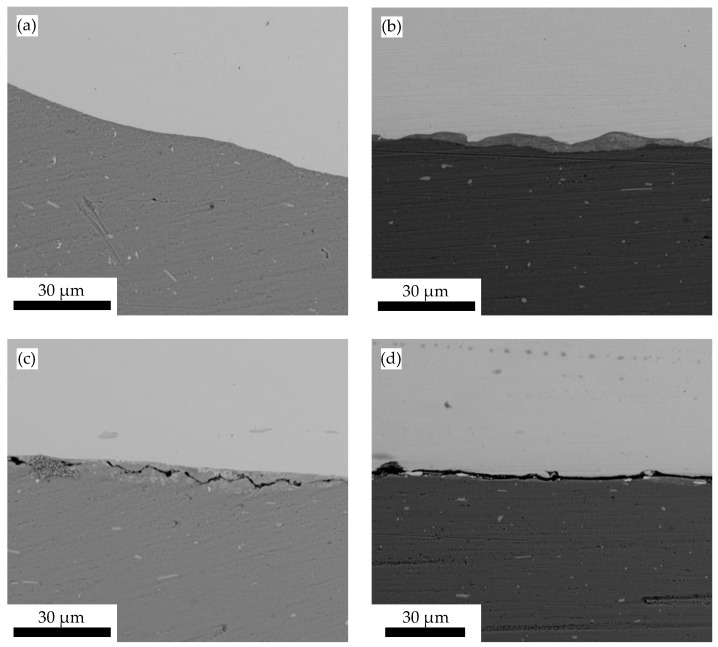
SEM images of the characteristic joint structures of the weld zone between steel DC04 (dark grey) and aluminium EN AW 6016-T4 (light grey): (**a**) weld zone without interlayer; (**b**) weld zone with interlayer (medium grey); (**c**) weld zone with cracked interlayer; (**d**) no contact between joining parts in the weld zone.

The investigations in the previous section show that the highest tensile shear strength values in the middle angular ranges of the welding process window are achieved for the weld joints produced at a collision speed of 349 m/s. In order to obtain a more precise statement about the tensile shear strength over the entire angular range of the welding process window at 349 m/s, further investigations are carried out. For this purpose, welded specimens from the entire angular range are first metallographically prepared according to the procedure described in Section 2. The weld zone of the prepared specimens is then examined by SEM. If different joint structures are observed along the entire length of the weld zone of the cut specimens, the position and length of the corresponding areas are measured; see Figure 2. These values are used to extrapolate the area ratios of the different joint structures to the total area A_w_ of the welded joint. If the length ratios of the structures are unequal on both sides, the area is calculated using non-square geometries such as a trapezoid. The following Section 3.4 presents the evaluation of the tensile shear strength values in relation to the extrapolated area fractions of the joint structures in the weld zone. Four characteristic joint structures are observed when the weld zones are examined by SEM, as shown in Figure 5 and briefly explained below. 

Figure 5a shows the characteristic joint structure of a weld without an interlayer. The steel material DC04 (dark grey) and the aluminium material EN AW 6016-T4 (light grey) lie directly on top of each other. The transition between the two materials in this structure is very smooth and no voids are visible. The SEM image in Figure 5b shows the structure where there is an interlayer (medium grey) between the aluminium material and the steel material. This area differs from the two base materials in both colour and structure. The interlayer is much smoother on the side facing the steel material (the bottom in the image) than on the top. In contrast, the top of the interlayer, i.e., the side facing the aluminium material, has a much more uneven or wavy surface. Similar to the structure shown in Figure 5a, no voids are visible. The thickness of the interlayer varies from 5 µm to 50 µm. However, the layer thickness is predominantly in the range of 10 µm. In the SEM images, lighter streaks or more sharply defined lighter areas can be seen in the interlayer, the colour of which is similar to that of the aluminium material. In addition, the interlayer resembles the aluminium–steel solid solution structure shown in [33]. For this reason, the structure shown here is assumed to be an aluminium solid solution.

The structures shown in Figure 5c,d both have voids of different shapes. The structure shown in Figure 5c will be referred to in the following as a weld zone with a cracked interlayer. The cracks occur only in the interlayer and not in the two base materials. The dimensions and optical composition of the interlayer correspond to the crack-free state shown in Figure 5b, so that an aluminium solid solution is also assumed here only in the cracked state. In addition, the SEM examination of the weld zones in some of the specimens reveals areas where there is no bond or direct contact between the two base materials. Figure 5d shows an example of such an area.

### 3.4. Tensile Shear Strength as a Function of Collision Angle

**Figure 6:**
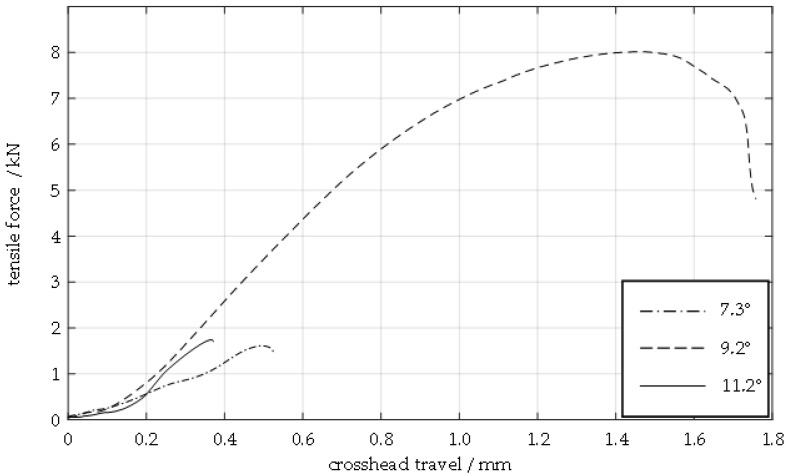
Force–travel diagram showing crosshead travel and tensile force in the tensile shear test for the material combination EN AW 6016-T4 (flyer, thickness: 1.5 mm) and DC04 (target, thickness: 2.0 mm) for a collision speed *v_imp_* of 349 m/s in dependence of the collision angle *β*.

As described in the previous section, the tests for the collision speed of 349 m/s are carried out on a significantly larger number of specimens, covering the full angular range of the process window. The diagram in Figure 6 shows the curve of tensile force and crosshead travel determined with the combined tensile and compression testing machine for three specimens welded at different collision angles *β* at a collision speed *v_imp_* of 349 m/s. The specimens with a collision angle of 7.3° and 11.2° fail after a short travel distance of the testing machine due to slipping in the welding zone. The specimen with a collision angle of 9.2°, i.e., in the centre of the process window, can withstand a significantly higher maximum force. This results in failure of the welded joint due to shearing in the weaker aluminium material; see Figure 4d,e.

**Figure 7:**
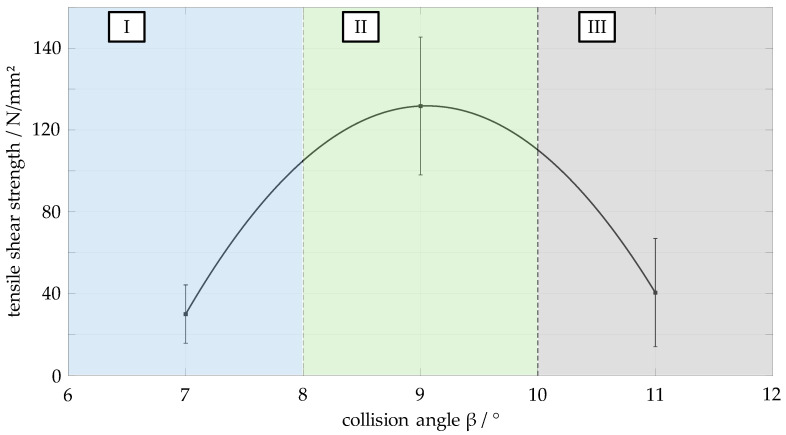
Tensile shear strength of the material combination EN AW 6016-T4 (flyer, thickness: 1.5 mm) and DC04 (target, thickness: 2.0 mm) in the angular areas for a collision speed *v_imp_* of 349 m/s.

The tensile shear strength and area ratios (see Section 3.5) of the joint structures present in the weld zone are determined for a total of 27 specimens. These 27 specimens divide the process window into three angular areas. Area I covers the angle range 6–8°, area II covers the angle range 8–10° and area III covers the angle range 10–12°. Figure 7 shows the mean values of the tensile shear strength determined for the three areas. Area II has the highest tensile shear strength with a mean value of 131 N/mm^2^. The strength values drop sharply towards the lower and upper limits of the process window, i.e., area I and area III, respectively. For example, a mean tensile shear strength of 40 N/mm^2^ is measured in area III, while area I has the lowest mean value of 30 N/mm^2^. The parabolic line shown in the figure is an interpolated trend line through the points of the three mean values determined.

### 3.5. Area Ratios of the Joint Structures as a Function of Collision Angle

**Figure 8:**
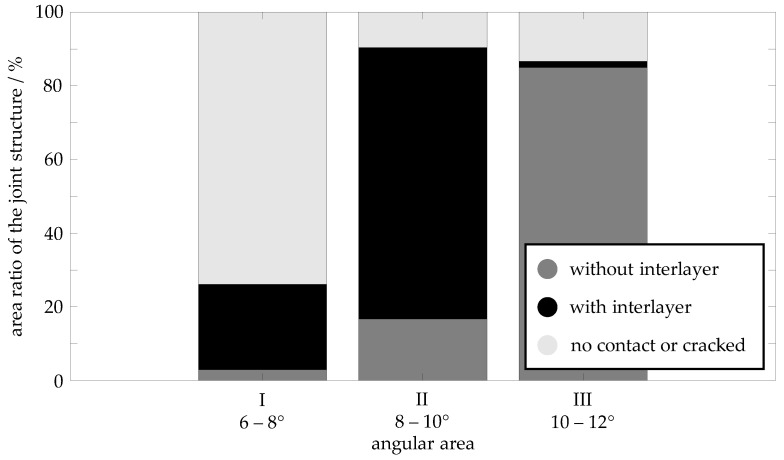
Area ratios of the joint structures of the material combination EN AW 6016-T4 (flyer, thickness: 1.5 mm) and DC04 (target, thickness: 2.0 mm) in the angular areas for a collision speed *v_imp_* of 349 m/s.

By analysing the SEM images of the 27 samples, the area fractions of the compound structures shown and described in Section 3.3 are determined using the procedure described there. The results of the analysis are shown in the diagram in Figure 8. In the diagram, the two joint structures weld zone with cracked interlayer (Figure 5c) and no contact between joining parts in the weld zone (Figure 5d) are combined into the superordinate structure no contact or cracked, as neither structure can transfer any forces in the weld zone between the aluminium and steel materials. Different joint structures dominate depending on the angle area. In area I (6–8°), the no contact or cracked joint structure has the largest area ratio at 73%, whereas in areas II (8–10°) and III (10–12°), it has an area ratio of only 10% and 13%, respectively. In area II, the weld zone with interlayer (Figure 5b) joint structure dominates with 73%. In contrast, it has a much lower proportion of 23% in area I and a strikingly low proportion of 2% in area III. Area III is dominated by the weld zone without interlayer (Figure 5a) joint structure. Its area ratio increases from 3% in area I to 17% in area II and 85% in area III.

## 4. Discussion

The results presented in this paper show, for a collision speed *v_imp_* of 349 m/s, a correlation between the collision angle *β*, the shear strength determined in tensile shear tests and the joint structures in the weld zones determined from SEM images. In the angle range near the lower limit (6–8°) of the welding process window, the largest area fractions in the weld zone are determined for the joint structures summarised in the superordinate structure no contact or cracked (see Figure 5c,d). This correlation can be attributed to the highly dynamic phenomena in the closing collision gap during the collision. Small collision angles lead to the highest collision point velocities *v_c_* (see Equation (1)), i.e., the fastest closing collision gap. In particular, unevenness and irregularities on the surfaces of the joining partners lead to strong turbulence. These impede the exit of the jet and particle cloud from the closing collision gap and lead to gas entrapment in the weld zone [23]. These entrapments are visible in the SEM images in the form of the observed cracked interlayer or in the form of areas of no contact between the base materials.

In the medium angle range (8–10°), the lower collision point velocity ensures that the jet and particles exit the closing collision gap, significantly reducing the area of cracked and unconnected structures. This can also be observed in the upper angular range. In addition, due to the conditions in the collision gap (pressure, compression of the medium in the collision gap, gas friction due to the escaping jet, etc.), the temperatures in the collision gap in the medium angular range are sufficiently high for a sufficiently long time to exceed the melting temperature of the base materials and melt them. The melting leads to the formation of a weld zone with an aluminium solid solution as an intermediate layer (see Figure 5b and [33]). At the upper end of the angle range (10–12°), there is not enough heating in the collision gap to cause the base materials to melt. The result is a smooth joint structure with no interlayer between the two base materials (see Figure 5a).

A comparison of the tensile shear strength values determined in relation to the surface areas of the four joint structures shows that the welded joints have the highest strength values in the middle angle range, although the described joint structure with an interlayer of aluminium solid solution dominates there. It is assumed that the strength values of the aluminium solid solution are lower than those of the two base materials. The high shear strength values of the joint are explained by the increase in surface area due to the wavy interlayer through which the solid solution is locally strongly interlocked with the two base materials. The occurrence of a wavy microstructure is also associated with favourable joint properties in terms of strength in [34,35], among others.

However, the correlation of the shear strength values with the surface areas of the four joint structures must always be considered against the background that the area shares determined are an extrapolation. The extrapolation is based on the analysis of two cut surfaces for each welded specimen using SEM images. It is not possible to accurately determine the area fractions between the two cut surfaces analysed using this method. For example, voids that are only present in the central area of the specimen and are not visible in the SEM images will reduce the tensile shear strength values determined, as these areas will also be incorrectly assumed to be joined.

Furthermore, the comparison of the observed joint structures with the schematic representation of the characteristic areas of different joint structures in the process window from [36] shows that the formation of the smooth joint structure without an intermediate layer (see Figure 5a) occurs in the upper angular range of the process window. In [36], this region shifts to larger collision angles *β* with increasing collision point velocity *v_c_*. The low tensile shear strength of the specimens with this joint structure of 40 N/mm^2^ on average (see Section 3.4) is attributed to the fact that the collision velocity *v_imp_* of 349 m/s is at the lower limit of the occurrence of this joint structure. It is expected that an increase in collision velocity will increase the welding process window for the material combination of EN AW 6016-T4 and DC04, particularly in the direction of the upper limit angle. Based on the statements in [36], it is expected that at higher collision speeds for the upper angle range, the specimens with a smooth joint structure without an intermediate layer will have higher tensile shear strengths.

It is not possible to verify the expected results described above with the current model test rig for collision welding as the maximum collision speed is limited to 349 m/s by design. A planned extension of the model test rig should allow a maximum collision speed of 500 m/s. The approach presented and successfully applied in this paper to correlate the shear strength with the percentage area of the different joint structures must be carried out in the future with a significantly larger sample size in order to reduce the influence of strength scatter. In addition, the angular areas need to be much smaller. In the context of the electromagnetic pulse welding (EMPW) process, it is therefore possible to simulate the transient behaviour of the collision angle and speed and use this approach to draw conclusions about the strength of the resulting welded joint.

## 5. Conclusions

This study comprehensively investigates the collision welding process for the material combination DC04 steel and EN AW 6016-T4 aluminium. Using a mechanical collision welding test rig, different collision angles are analysed to determine their effect on the formation of the joint structure of the weld zone and the tensile shear strength of the welded joints. The results show that medium collision angles (8–10°) at a collision speed of 349 m/s produce the most favourable welding conditions, characterised by minimal cracking and unconnected structures, resulting in higher tensile shear strength. In further research, the interlayer in the weld zone, which varies in appearance depending on the collision angle, will be analysed by EDX analysis in order to determine the metallurgical composition of this layer as a function of the collision angle. The planned extension of the model test rig to collision speeds of 500 m/s will allow the potential further increase in strength of the welded joint at higher collision speeds to be investigated in the future.

## Data Availability

The original contributions presented in the study are included in the article, further inquiries can be directed to the corresponding author.
